# Combined Effect of Abusive Supervision and Abusive Supervision Climate on Employee Creativity: A Moderated Mediation Model

**DOI:** 10.3389/fpsyg.2020.01175

**Published:** 2020-07-10

**Authors:** Chuangang Shen, Jing Yang, Sanman Hu

**Affiliations:** ^1^School of Business, Huaqiao University, Quanzhou, China; ^2^College of Tourism, Huaqiao University, Quanzhou, China

**Keywords:** employee creativity, abusive supervision, abusive supervision climate, creative role identity, multilevel analysis

## Abstract

In accordance with social identity theory, a multi-level model is put forward to investigate how the “conjoint” associations between abusive supervision and abusive supervision climate exert influence on employee creativity through creative role identity. The data in this paper were from 357 supervisor-subordinate dyads in 77 working groups to test the proposed model. The results indicated that creative role identities mediated the relationship between abusive supervision and employee creativity, and group-level abusive supervision climate moderated the relationship between creativity and individual-level abusive supervision through the process of creative role identity, the mutual influence of abusive supervision climate and individual-level abusive supervision significantly predicated employee creativity. This paper also discusses related managerial and practical implications.

## Introduction

The development of a “knowledge economy” and extensive competition for businesses have motivated organizations and managers to place greater emphasis on incentive mechanisms to stimulate employee creativity (Dong et al., 2017; Dong Liu et al., 2017), which means the relationship between leadership and creativity has become a hot research topic in organizational behavior field (Zhou and Hoever, 2014). A number of preceding studies put their focus on the positive influence that leadership can have on the promotion of an employee’s creativity, such as transformational leadership (Gong et al., 2009), empowering leadership (Harris et al., 2013; Zhang et al., 2018), authentic leadership (Cerne et al., 2013), benevolent leadership (Wang and Cheng, 2010; Lin et al., 2018), charismatic leadership (Murphy and Ensher, 2008), moral leadership (Gu et al., 2015), and so on. More recently, with the emergence of research on negative leadership styles, such as abusive supervision, a series of studies have been conducted to explore its influence on employee creativity (Liu et al., 2012; Lee et al., 2013; Zhang et al., 2014; Han et al., 2017). Nevertheless, their results are inconsistent. Some researchers found that employee creativity is impacted negatively by abusive supervision (Liu et al., 2012; Zhang et al., 2014; Han et al., 2017), while others have demonstrated that an inverted U effect is imposed on employee creativity by abusive supervision (Lee et al., 2013). These inconsistent research findings are challenging organizations with regard to effectiveness of management guidelines and practice. In addition, the inconsistencies also indicate a complicated relationship between abusive supervision and employee creativity. Therefore, it is essential to think about the process and circumstances through which abusive supervision performs its influential power on employee creativity.

Previous studies have emphasized internal motivation function and psychological safety to elaborate on how abusive supervision exerts influence on employee creativity (Zhang et al., 2014; Liu et al., 2016), and have neglected examining the effect of creative role identity, despite scholars generally recognizing that creative role identity performs a significant role in promoting employee creativity. Creative role identity represents whether an individual regard himself/herself as a person with powerful creativity (Farmer et al., 2003; Tierney and Farmer, 2011). According to identity-based motivation (Oyserman, 2007), creative role identity that can enhance individual internal motivation is a relatively fundamental factor in driving individual’s creativity. Meanwhile, the researchers and scholars place more and more attention on the viewpoint of follower-based leadership, in which leaders exert a subtle influence on their followers via their influential power on an individual’s self-concept. Some researchers demonstrated that a supervisor can affect the cognition, affection, and behavior of his/her subordinates by effectively changing the subordinates’ self-perspective during supervisor-subordinate interaction processing (Luo et al., 2016). It manifests that creative role identity may be playing a mediating role in the associations between employee creativity and leadership. Therefore, in current study, we intend to explore the mediating role of creative role identity between employee creativity and abusive supervision.

**FIGURE 1:**
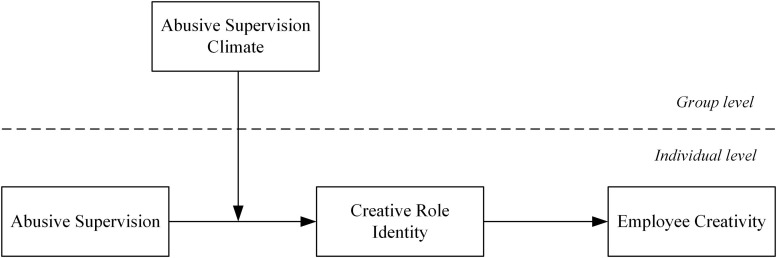
Proposed model.

A greater emphasis has been placed on supervisory abuse as a personal-level phenomenon in the current research field of abusive supervision (Lee et al., 2013; Liu et al., 2016). However, working teams have become the smallest unit within organizations presently. It is more likely that abusive supervision will happen in public instead of “in a vacuum” (Liao et al., 2018), so team members know clearly about the abusive treatment coming from an individual leader. A common perception of supervisory abuse against team members is easily shared among the same working group, such unfavorable ambience being referred to as an abusive supervision climate (Peng et al., 2014; Priesemuth et al., 2014). The abusive supervision climate within a working team provides spaces for comparing abusive treatments which team members once suffered (Jiang et al., 2017). In terms of social comparison theory, the individuals are inclined to make interpersonal comparison, particularly compare with members in the same group (Festinger, 1954). Under disadvantaged circumstance, one’s personal perception of destructive treatment is normally on the basis of the “miserable” treatment suffered by others in their neighborhood because other people’s similar negative treatment from same cause lay down the foundation for social comparison (Gerber et al., 2018). Consequently, social comparison results will either strengthen or weaken employees’ response to negative treatment. Therefore, we put forward that individual-level abusive supervision and abusive supervision climates may impose conjunct effects on employees’ responses, particularly the associations between individual-level abusive supervision and employees’ creative role identity may be subject to influence of an abusive supervision climate. Figure 1 presents our hypothesized model.

Our study may be help to promote the advancement of organizational behavior literature and practice from three important aspects. First, the study validates an integrated model incorporating the concepts of an abusive supervision climate, creative role identity, abusive supervision and employee creativity from the perspective of role identity, providing a better understanding of abusive supervision being a multi-level matter and further deepening our insights into why employee creativity may not be impaired in an unfriendly working atmosphere. This study also provides a possible explanation for previous inconsistent findings, and enriches the literature on abusive supervision. Second, the study treats creative role identity as a kind of mediating mechanism in the relevant relationship between employee creativity and abusive supervision. It will deepen our insights of the process, and how abusive supervision exerts its influence on employee creativity and provides a new interpretation framework from a role identity perspective, which expands the literature on employee creativity, such as the influence factors and mechanism. Furthermore, this study will provide practical insights for organizational managers who hope to promote employee creativity by eliminating negative factors that may impair employee creativity.

## Theories and Hypotheses

### Abusive Supervision and Employee Creativity

Employee creativity can be defined as the generation of beneficial unprecedented thoughts with regard to services, products, commodities, practical implementation, or processing procedures at workplace (Stobbeleir et al., 2011). Creativity research debated that the creation of novel thoughts requires considerable investment of efforts and time and involves various processes (Feng et al., 2018). Leadership is a critical contextual factor for driving employees to think unconventionally at work and motivating employees to invest a significant amount of time and energy in the process of innovation (Hirst et al., 2009). Showing support, care, encouragement and other positive leadership behaviors in supervisor–subordinate interactions benefits employees, and recognizing their supervisors’ open attitude toward new ideas and thoughts encourages them to break the routine, to change the *status quo*, and devote themselves to innovative tasks with greater investment of time and efforts (Zhang and Bartol, 2010; Dong et al., 2017; Zhang et al., 2018). Abusive supervision is defined as “the extent to which supervisors engage in the sustained display of hostile verbal and non-verbal behaviors, excluding psychical contact” (Tepper, 2000), such as receiving cynical comments or being mocked that their ideas are silly or they are incompetent (Tepper, 2000; Tepper et al., 2017). The emotional feelings of being offended, or being scorned by their supervisor force employees to question their own contributions to the progressive development of the organization, their competent capabilities, and the significance of the work, which may overwhelmingly weaken their positive devotion in the process of innovation, particularly when it comes to the investment of efforts and time. This unfavorable situation may even inhibit their organization-benefiting behavior. Therefore, under the context of abusive supervision, employees are less likely to accept an innovative approach or innovative thoughts for trouble-shooting. According to this analysis, the following hypothesis is developed in this study:

**Hypothesis 1:** abusive supervision is negatively related to employee creativity.

### Mediating Effect of Creative Role Identity

Creative role identity is defined as self-identification of being a creative person, an individual regards such identity as an utmost important component of his/her duties (Farmer et al., 2003). It is about the degree to which an individual defines himself/herself as a person with certain creative power (Farmer and Tierney, 2017). According to role identity theory, the sense of creative role identity is based on self-related feedback or role expectation acquired from social relationships, especially the role expectation presented in the response of feedback from critical social connections constitutes a key factor in influencing the formation of a substantive role identity of an individual (Derue and Ashford, 2010). Supervisors are a primary source for providing feedback related to employees in the workplace, and when interacting with subordinates, supervisor’s behaviors are the most direct representation of his/her feedback for the subordinates regarding role expectations (Derue and Ashford, 2010; Epitropaki et al., 2017). When a supervisor demonstrates positive leadership behaviors in an interaction with subordinates, such as acknowledging employees’ contributions and encouraging employees to employ innovative approaches for trouble-shooting, the employees will realize that their supervisor has higher creative role expectation of them, and this conscious awareness facilitates the development of employees’ creative role identity (Wang et al., 2014; Qu et al., 2015). However, when a supervisor belittles, suppresses, or ridicules his/her subordinates, suggesting that the supervisor has a lower creative role expectation of his/her subordinates, this will force the employees to doubt their own creative perspectives, beliefs, and thoughts, thereby hindering the development of high creative role identity (Mackey et al., 2017).

In addition, according to role identity theory, identification of an individual with a specific role drives him/her to display the behaviors of such specific role; the more convinced an individual is of a self-perceived role, the better aligned the individual’s behavior is with that specific role identity (Kaplan and Garner, 2017). An employee with a strong creative role identity will believe that generating innovative and useful ideas or viewpoints is a core component of his/her work. Such a type of employee will stay highly sensitive and adaptive to the external environment. In order to promote in-depth thinking and surpass the expectation of the required role identify, those employees are willing to invest considerable efforts and time to develop innovative trouble-shooting strategies, and as a result, they present strong creativity. By contrast, employees with weaker creative role identity do not regard themselves as highly creative individuals, and when it comes to trouble-shooting at work, they do not dedicatedly seek out novelty for trouble-shooting. Such apathy is detrimental for creativity. In terms of related analysis, this study considers that when employees are abused by their supervisors, the development of their creative role identity will be suppressed, making them reluctant to invest efforts and time in creative activities. Therefore, the following hypothesis is established in this study.

**Hypothesis 2:** Creative role identity mediates the effect of abusive supervision on employee creativity.

### Moderating Effects of Abusive Supervision Climates

Among a team group, a certain discrepancy may arise when a leader interacts with his/her group members, causing employees’ self-value, role, and other self-perspectives in the group to differ (Epitropaki et al., 2017; Gerber et al., 2018). The differentiated treatment of leaders provides space and chances for social comparison among team members (Tse et al., 2013). The result of social comparison will either strengthen or weaken the employees’ self-perspective after receiving individual-specific treatment (Strickhouser and Zell, 2015). According to role identity theory, the forming of self-meaning through role identity is a process of self-regulation in which coordinated information inputs from others and oneself substantiate the identity (Farmer et al., 2003). In other words, during the process of forming its role identity, it may inevitably make social comparison with people nearby, because social comparison is also an important means to obtain information about themselves (Festinger, 1954). Therefore, it is unavoidable in a workplace that individuals compare themselves with other group members who have a similar standing to accurately assess their competence, attitude, self-value, and the relative position in the group. It means that an individual’s self-evaluation will strengthen (or weaken) if the individual considers his/her experience is better (or worse) than others’ (Farh and Chen, 2014; Strickhouser and Zell, 2015; Jiang et al., 2017). In the abusive supervision context, for group members, an abusive supervision climate within the group acts as a benchmark for comparison. A group with a relatively low level abusive supervision climate means that most of group members have experienced a relatively low level of abuse. When a single employee perceives a relatively high level of abuse, he/she will feel like they are being “singled out,” and through social comparison, he/she will detect that other colleagues are not abused as he/she is. In this case, the negative influence of the individual-level abusive supervision imposed on the employee’s creative role identity will be exacerbated (Farh and Chen, 2014).

In contrast, in a group with a relatively strong abusive supervision climate (i.e., the group members experience a high level of abuse generally), when a focused member perceives a high level of abuse, this focused member will feel the experienced abuse is similar to what other group members have experienced. In this case, the individual member will not make favorable or unfavorable social comparison (Hu and Liden, 2013). Moreover, the individual member may recognize that he/she is neither better off nor worse off than others, so the effect of abusive supervision on creative role identity weakens. However, even if the level of abuse experienced by an individual is not higher than what other members of same group have experienced, the individual may still have a relatively weaker creative role identity similar to those who had experienced higher level of abusive supervision. This is because the observance of seeing the supervisor abusing other group members may cause individuals to generate social anxiety-like negative emotions and make each of them believe that they will be the next abused victim of the supervisor (Jiang et al., 2017). Consequently, all employees within a same group perceive a low creative role expectation from their supervisor and uniformly consider themselves not as a highly creative individual. Therefore, in a high abusive supervision climate, all members of the group may perceive a relatively weak creative role identity. The following hypothesis is established in this study:

**Hypothesis 3:** An abusive supervision climate can moderate the negative effect of individual-level abusive supervision on creative role identity. The negative relevance between individual-level abusive supervision and creative role identity is stronger when the level of abusive supervision climate is lower and weaker when the level of abusive supervision climate is higher relatively.

This study proposed an integrated, moderated mediation model based on Hypotheses 2 and 3. Concretely speaking, when a member of a group with an abusive supervision climate experiences higher-than-average individual-specific abuse, this individual will feel that the supervisor has a low innovative role expectation of him/her, which reduces the individual’s creative role identity and makes the individual reluctant to invest a great amount of time and efforts into innovative activities. This situation is unfavorable for enhancement of employee creativity. In a group with a high abusive supervision climate, regardless of how individuals perceive the individual-level abuse, all group members will feel that their supervisor does not support innovations and has a low expectation on group members for innovations. In this case, employees’ involvement in creative activities will decline. This study therefore develops the following hypothesis:

**Hypothesis 4:** An abusive supervision climate moderates the mediation of creative role identity on the relationship between individual-level abusive supervision and employee creativity; specifically, the mediated relationship is weaker in high abusive supervision climates than that of in low abusive supervision climates.

## Method

### Procedure and Samples

A total of 470 employees from 77 working groups in 17 companies of a corporation in China were involved in this study: six groups from one company, five groups from ten companies, four groups from three companies, and three groups from the last three companies. Before conducting the survey, we sought the cooperation of the human resources departments and randomly selected working groups including supervisors and their subordinates. All the participants were informed that the objective of this research is to investigate the leadership style and dynamics of interaction within groups in the company and were informed that the investigation was voluntary. If a participant completes the survey, it means informed consent. The researchers also emphasized that the investigation was anonymous before handing out questionnaires. To effectively control common method bias, aside from collecting data using supervisor-subordinate dyadic, questionnaires filled out by subordinates were scored by different scales. Subordinates were required to evaluate abusive supervision, the abusive supervision climate and creative role identity, while supervisors were requested to evaluate creativity of their subordinates. To ensure that subordinates provided truly reliable information when answering the questionnaire, we encased each subordinates’ questionnaire into a sealed envelope with double-sided tape, also remind them to seal the envelope before returning to the researchers. All the questionnaires were coded to match a subordinate’s response with their supervisor’ creativity scoring.

Dyad-matched questionnaires were delivered to 470 subordinates and 77 corresponding supervisors. A total of 433 supervisor questionnaires were retrieved (92.1%), while 407 subordinate questionnaires were retrieved (86.6%). After validation, 357 supervisor-subordinate dyad questionnaires were collected. In this study, the percentage of male participants is 54.6%. The average age of all subordinates is 29.06 years. The average year of the subordinates having been working for their current company and having been working together with their current supervisor is 6.51 and 2.73 years, respectively.

### Measures

Since the research variable measurements used in our study were developed in Western countries, a translation and back-translation procedure were employed to assure the validity and reliability of the Chinese-versioned scales.

#### Abusive Supervision Climate

Using the five-item scale once adopted by Priesemuth et al. (2014), the abusive supervision climate was assessed accordingly. The subordinates were requested to evaluate their acceptable agreement with question statements using a 5-point response format (1 for completely disagree, 5 for completely agree). For example, questions such as “My supervisor ridicules my colleagues in same work group,” “My supervisor scorns my colleagues in same work group that they are incompetent.” Cronbach’s alpha for the abusive supervision climate scale in this study is 0.84.

By following previous climate research, in order to conclude a final group-level abusive supervision score, individual values for the abusive supervision climate across all team members in a same work group are averaged. In order to examine the feasibility of intra-group abusive supervision climate aggregation, we have examined R_wg_ and intra-group correlation between *ICC*(1) and *ICC*(2). The related scorings of the abusive supervision climate is 0.96 (R_wg_ s), 0.37 [ICC(1)], and 0.73 [ICC(2)], respectively. According to James (1982), R_wg_ should be greater than 0.7, whereas ICC(1) and ICC(2) should be greater than 0.05 and 0.5, respectively. Therefore, an averaged score of the abusive supervision climate perceived by individuals within a same group could be used as an index to assess the intra-group abusive supervision climate.

#### Abusive Supervision

Using a ten-item scale constructed by Aryee et al. (2007), abusive supervision was assessed. The applied ten-item scale is a revised version of the original fifteen-scale developed by Tepper (2000). Using a 5-point response format (1 for completely disagree, 5 for completely agree). Subordinates were requested to rate their acceptable agreement with statements like “My line leader mocks my thoughts or feelings as foolish” and “My line leader speaks evil of me behind my back to others.” Cronbach’s alpha for the abusive supervision scale in this study is 0.94.

#### Creative Role Identity

Using a three-item scale constructed by Farmer et al. (2003), creative role identity was assessed. Participants were requested to rate their acceptable agreement with the statements using a 5-point Likert response format (1 for completely disagree, 5 for completely agree), like “I often consider creativity” and “Being a creative employee is a crucial part of my identity.” Cronbach’s alpha for the creative role identity scale in this study is 0.77.

#### Employee Creativity

Using the four-item scale constructed by farmer et al., employee creativity was assessed. The supervisors were requested to rate their acceptable agreement with the statements using a 7-point Likert response format (1 for completely disagree, 7 for completely agree), like “this employee is first one to make an attempt to new ideas or methods” and “this employee tries to find new ideas and ways for trouble-shooting.” Cronbach’s alpha for employee creativity scale in this study is 0.85.

#### Control Variables

Previous studies have suggested that some demographic variables affected individual creativity, such as age (Binnewies et al., 2008), the gender of the employees and supervisors (Stoltzfus et al., 2011), and tenure (Ng and Feldman, 2013). Hence, in this study, employee’s age, company, gender of employee and their supervisors, tenure, number of years working with their supervisor were controlled.

## Results

### Analysis Strategy

A hierarchical linear model (HLM) was employed to examine the multi-level model. A two-level HLM was constructed in which group members were specified at level 1 and teams at level 2. In accordance with recommendations suggested by Preacher et al. (2007), we examined group-mean centered individual-level variables and grand-mean centered group-level variables (abusive supervision climate). Complying with the procedure proposed by Baron and Kenny (1986), we examined the main effect (H1) and the mediating effect (H2). For examining the cross-level moderating effect (H3 and H4), we adopted recommendations of Muller et al. (2005) and have validated Hypotheses 3 and 4 using the following equations. Hypothesis 3 (mediating effect) suggests that the impacts of individual-level abusive supervision on creative role identity depends on the abusive supervision climate. Hypothesis 4 (moderated mediation) suggests that the mediating effect that individual-level abusive supervision has on employee creativity through creative role identity depends on the abusive supervision climate. Therefore, both individual-level abusive supervision and the interaction between individual-level abusive supervision and an abusive supervision climate can impose significant impacts on employee creativity. Moreover, the impacts of creative role identity on employee creativity or the impacts of the interaction between an abusive supervision climate and creative role identity should be significant on individual creativity. In the analysis here, the centralized overall mean is adopted for the group-level variable (abusive supervision climate), whereas the centralized group mean is adopted for the individual-level variables.

### Confirmatory Factor Analysis

Before the hypotheses had been tested, Amos 24.0 was used to perform confirmatory factor analysis on four variables to test the discriminative validity. Table 1 showed related results. The hypothesized four-factor model (Model 5) with abusive supervision climate, abusive supervision, creative role identity, and employee creativity presented a contented goodness of fit [χ^2^(203) = 301.24, *p* < 0.01, df = 203, GFI = 0.93, CFI = 0.98, RMSEA = 0.04, TLI = 0.97], and the four-factor model performed better for data fitting than other models, like three-factor, the two-factor, or the one-factor model. Therefore, our study realized the discriminant validity successfully.

**TABLE 1:** Confirmatory factor analysis results for hypothesized variable (*N* = 357).

**Model**	**χ^2^**	**df**	**χ^2^/df**	**RMSEA**	**GFI**	**CFI**	**TLI**
Model 1: one-factor	1346.21	209	6.44	0.12	0.69	0.74	0.71
Model 2: two-factor	814.25	208	3.91	0.09	0.79	0.86	0.85
Model 3: three-factor^a^	592.20	206	2.87	0.07	0.84	0.91	0.90
Model 4: three-factor^b^	528.00	206	2.56	0.07	0.87	0.93	0.92
Model 5: four-factor	301.24	203	1.48	0.04	0.93	0.98	0.97

*Model 1: abusive supervision + abusive supervision climate + creative role identity + creativity; Model 2: abusive supervision + abusive supervision climate, creative role identity + creativity; Model 3: abusive supervision + abusive supervision climate, creative role identity, creativity; Model 4: abusive supervision, abusive supervision climate, creative role identity + creativity; Model 5: abusive supervision, abusive supervision climate, creative role identity, creativity.*

## Descriptive Statistics and Correlation Analysis

In Table 2, every variable’s mean, standard deviation, and correlation coefficient is shown. According to the results shown in Table 2, there is a significantly negative relevance between abusive supervision and employee creativity, also between abusive supervision and employee creative role identity. However there also exists a significantly positive relevance between employees’ creative role identity and creativity.

**TABLE 2:** Descriptive statistics and correlations.

	***M***	**SD**	**1**	**2**	**3**	**4**	**5**	**6**	**7**	**8**
**Level 1**										
1. Company	–	–	1.00							
2. Gender of supervisor	1.55	0.50	–0.09	1.00						
3. Gender of employee	1.45	0.50	−0.32**	0.36**	1.00					
4. Age	29.06	6.46	–0.08	–0.02	0.26**	1.00				
5. Tenure	6.51	5.89	−0.19**	–	0.29**	0.61**	1.00			
6. Time working with supervisor	2.73	2.37	0.02	0.02	0.23**	0.43**	0.54**	1.00		
7. Abusive supervision	1.96	0.83	0.17**	–0.07	0.05	0.08	0.10*	0.17**	1.00	
8. Creative role identity	3.46	0.88	–0.10	0.06	–0.05	–0.02	–0.01	–0.07	−0.42**	1.00
9. Creativity	4.91	0.92	−0.13*	0.03	–0.01	–0.03	–0.02	–0.04	−0.32**	0.41**
**Level 2**										
1. Abusive supervision climate	1.63	0.35								

*N* = 77 (supervisors); *N* = 357 (employees). Gender was coded “1” for men and “2” for women. *p < 0.05, **p < 0.01.

### Hypothesis Testing

Hypothesis 1 suggests that a negative relevance exists between employee creativity and individual-level abusive supervision. Indeed, as indicated in Table 3 (M3), the negative influence of abusive supervision on employee creativity was significant (γ = −0.33, *p* < 0.01), which supported Hypothesis 1.

**TABLE 3:** Hierarchical linear modeling Regression results.

	**Creative role identity**		**Creativity**			
	**M1**	**M2**	**M3**	**M4**	**M5**	**M6**
**Level 1 (N = 357)**						
Company	−0.04(0.06)	–	−0.09*(0.05)	−0.08(0.05)	−0.09(0.07)	−0.07(0.06)
Gender of supervisor	−0.09(0.12)	−0.05(0.11)	−0.03(0.13)	−0.01(0.12)	−0.01(0.13)	−0.04(0.12)
Gender of employee	0.07 (0.09)	0.07 (0.09)	0.02 (0.09)	–	0.03 (0.08)	0.02 (0.09)
Age	–	–	–	–	–	–
Tenure	0.01 (0.01)	0.01 (0.01)	−0.01(0.01)	−0.01(0.01)	–	–
Time working with supervisor	–	−0.01(0.02)	0.02 (0.02)	0.02 (0.02)	0.02 (0.02)	0.01 (0.03)
Abusive supervision	−0.43**(0.06)	−0.38**(0.07)	−0.33**(0.05)	−0.20**(0.06)	−0.35**(0.08)	−0.32**(0.10)
Creative role identity				0.30**(0.05)		0.20**(0.06)
**Level 2 (N = 77)**						
Abusive supervision climate		−0.36**(0.19)			−0.09(0.21)	–
Abusive supervision × abusive supervision climate		0.53*(0.21)			0.53*(0.23)	0.59*(0.29)
Abusive supervision climate × creative role identity						0.21 (0.19)

*p < 0.05, **p < 0.01.

We adopted the procedures devised by Baron and Kenny (1986) to test Hypothesis 2, which proposed that creative role identity may mediate the negative effects of abusive supervision and employee creativity. As shown in Table 3, the result of Model 2 indicated that there is a significant main effect of abusive supervision on creative role identity (γ = −0.43, *p* < 0.01). Meanwhile, when individual-level abusive supervision and creative role identity are both included into the equations to predict employee creativity, it was found that creative role identity can significantly predict employee creativity (γ = 0.30, *p* < 0.01). The predictive effect of individual-level abusive supervision on employee creativity is weaker than that of M2’, but still significant (γ = −0.20, *p* < 0.01). This result presents that employee creative role identity partially mediated the influence of abusive supervision on employee creativity. Therefore, Hypothesis 2 is supported.

**FIGURE 2:**
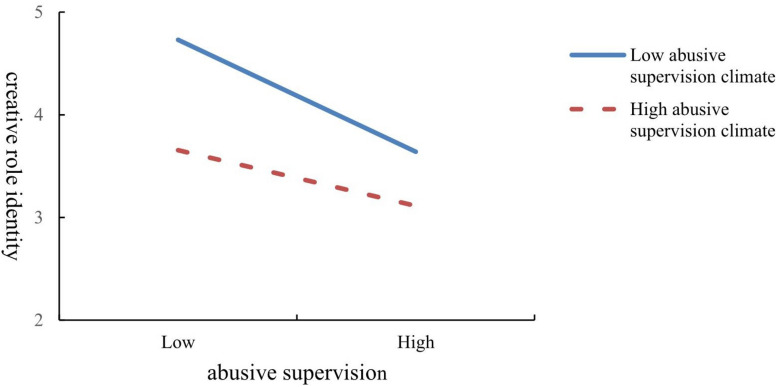
Interactive effect of abusive supervision climate and abusive supervision on creative role identity.

Hypothesis 3 predicts that the abusive supervision climate and individual-level abuse have a cross-level interaction on employees’ creative role identity. As shown in Table 3 (M2), we found that the interaction term between individual-level abusive supervision and an abusive supervision climate can significantly predict creative role identity (γ = 0.53, *p* < 0.01). For illustration of the relationship, Figure 2 shows the interaction pattern. In accordance with the recommendations of Aiken et al. (1991), Figure 2 has revealed one standard deviation above and below the mean of abusive supervision climate. Consequently, the result validated Hypothesis 3.

Hypothesis 4 predicts that an abusive supervision climate moderates the mediating effect of creative role identity between employee creativity and abusive supervision. According to suggestions from Muller et al. (2005), M5 in Table 3 indicates that the mutual effect between individual-level abusive supervision and abusive climate can significantly forecast employee creativity (γ = 0.53, *p* < 0.05). M6 indicates that even though the mutual effect between abusive supervision and creative role identity has failed to forecast employee creativity significantly (γ = 0.21, *p* > 0.05), creative role identity has significantly predicted employee creativity (γ = 0.20, *p* < 0.01), suggesting that creative role identity’s mediating effect on the associations between employee creativity and individual-level abusive supervision was alleviated by the abusive supervision climate. The result of simple effect analysis indicates that the indirect effect is −0.161 (*p* < 0.05) in a weak abusive supervision climate, while the indirect effect is −0.062 (*p* < 0.05) in a strong abusive supervision climate. Thus, Hypothesis 4 is supported.

## Discussion

The primary purpose of our study is to explore the insights into how and when abusive supervision harm employee creativity. The predominant theories, social role identity theory and social comparison theory, are selected as lenses of perspectives in the study which finds that abusive supervision can negatively affect employee creativity, and creative role identity mediates the associations between employee creativity and abusive supervision. Moreover, study results indicate that an abusive supervision climate can establish a cross-level contextual factor that may moderate the indirect effect of individual-level abusive supervision on employee creativity through creative role identity, which indicates that when the abusive supervision climate is low, individual-level abusive supervision will exert a strong negative impact on employees’ creative role identity, further weakening employee creativity. Nonetheless, the mediating effect is insignificant when the abusive supervision climate is strong. These findings present some interesting managerial and theoretical implications.

### Theoretical Implications

The study findings in this article contribute to research on creativity and abusive supervision from several aspects. First of all, by examining the associations between employee creativity and the “dark side” of leadership (abusive supervision) and uncovering abusive supervision’s inhibiting effect on employee creativity which were inconsistent in preceding research (Liu et al., 2012, 2016; Lee et al., 2013), this study is beneficial to the existing creativity literature. Therefore, this study can provide new evidence validating the negative correlation between employee creativity and abusive supervision. Furthermore, this study has supplemented new research information about abusive supervision and employee creativity research, addressing the inconsistent findings in preceding studies and positively supporting the proposition of Zhou and Hoever (2014), in terms of negative leadership increasing impacts on employee creativity.

Second, by providing a new interpretation framework, our findings also benefit the existing literatures on creativity and abusive supervision. Previous studies explained how employee creativity is subject to the influential effects of abusive supervision from the perspectives of internal motivation and psychological safety (Zhang et al., 2014; Liu et al., 2016), this study offers an interpretation of how employee creativity is influenced by abusive supervision from the perspective of the creative role identity. The research results displayed that employee creative role identity performs a crucial mediating role between abusive supervision and employee creativity. The new perspective and direction are proposed for understanding the associations between abusive supervision and employee creativity. Moreover, the research results have validated the propositions of Farmer et al. (2003), which creative role identity is a key driver to promote employee creativity. Farmer et al. (2003) put forward that creative role identity is critical for promoting employee creativity and may be a crucial factor in connecting leadership to employee creativity. Previous studies have tested the influence of creative role identity on the relationship between transformational leadership and employee creativity (Wang et al., 2014). This study showed that creative role identity influences the relationship between negative leadership and employee creativity. Whereas Liu et al. (2016) have called for examining the psychological mechanism of the effect of abusive supervision on employee creativity, this study has effectively deepened our insights about how employee creativity is impacted by abusive supervision and provided new direction for organizations implementing preventive measures.

Third, this study turns the focus to the group level and benefits integration of the role identity theory with the social comparison theory for testing the abusive supervision climate’s moderating effect on individual-level abusive supervision and creative role identity. Human-group interaction is examined in the study from the multi-level perspective. Concretely speaking, the group context cultivates group members’ abusive supervision experiences. The effect of being “singled out” presented in the findings of an earlier study has been supported by the results of this study. That is, the negative correlation between individual-level abusive supervision and creative role identity is strong in a weak abusive supervision climate, and so is the mediating effect of creative role identity on the relationship between abusive supervision and employee creativity. Nonetheless, an even more interesting finding of this study is about individuals being in a strong abusive supervision climate but experiencing only a minor degree of abuse. For these individuals, even though they are not the subject to the abuse of their supervisor, when compared to other individuals from a group with a weak abusive supervision climate they still show lower creative role identity, which is consistent with that of Farh and Chen (2014), demonstrating that when the group-level abuse is strong, individuals who are not the abuse object but are in the same abusive supervision situation will also experience a relatively low organization-based self-esteem (OBSE). The aforesaid study confirmed earlier studies’ inference on uncivilized behavior; the individuals witnessing but not experiencing uncivilized treatment share the same experience of those targeted individuals (Tepper, 2000; Jiang et al., 2017). In other words, a vicarious experience of the supervisor’s abusive supervision exists in those non-targeted individuals. For abusive supervision researchers, it means that they should take group-level contextual factors of abusive supervision climate into account to completely comprehend the process and impacts of abusive supervision.

### Practical Implications

The following practical implications are put forward in this study. First of all, the negative impacts of abusive supervision on employee creativity found here suggests that organizations and managers should recognize the “harm” of supervisors’ abusive supervisory behavior. Therefore, organizational measures, such as enhancing managers’ sensitivity toward abusive supervisory behavior, should be implemented, so that managers can effectively monitor individual-level supervisory behavior. At the same time, organizations should have zero tolerance for abusive supervision and establish related rules, regulations, and systems to effectively prevent the occurrence of abusive supervisory behavior and eliminate negative factors hindering improvement of employee creativity in the organization. Second, the group-level abusive supervision climate moderates the associations between creative role identity and individual-level abusive supervision perception, suggesting group leaders’ differential treatment to group members can result in a profound impact on employees’ self-perspective. This finding suggests that organizations should improve supervisors’ management skills, including interaction techniques and emotion management, enabling group members to perceive their leaders’ behavior as fair and appropriate. Third, this study found that employees’ creative role identity plays a critical mediating role between abusive supervision and employee creativity. This result reminds us that organization managers should identify employees with a strong creative role identity and provide them with job positions that enable them to present or improve their creative self-perspective, so these employees with a strong creative role identity are able to sustain their self-perspective. Moreover, organizations should develop related policies for supporting employees’ creative efforts, encouraging them doing so, and keeping an inclusive attitude toward employees when their innovative attempts fail, to positively maintain employees’ creative role identity. Thus, employees will be able to present high creativity at work.

### Limitations and Future Directions

Several limitations should be noted in this study. First of all, A cross-sectional design is employed in the study, which cannot support inferences of causality. Earlier researchers have found that in an organization, employees with poorer performance inferior to others tend to be subject to more abuse. Nevertheless, whether it is the supervisor’s abusive supervision that weakens employee creativity, or the employee’s low creativity at work provoking the supervisor’s abusive behavior, remains unclear. As a result, future studies can employ an experimental approach or conduct a longitudinal follow-up study to further explore the causality between abusive supervision and employee creativity. Second, this study benefits supervisors’ subjective rating of employees’ creativity to effectively avoid the effect of social desirability from employees’ self-evaluation. However, the effect of supervisors’ personal bias may exist. Therefore, future studies can employ more objective creativity measurements, such as innovative performance bonuses, proposals of innovative ideas, and number of patented inventions. Furthermore, although this study explored the impacts of abusive supervision on employee creativity from group level, the control variables were centered on the individual level, namely, group members. As a result, this study did not discuss group creativity. Considering that the improvement of group creativity has become a major concern in creativity research nowadays, future studies should examine the effect as well as the internal mechanism of group-level abusive supervision on group creativity to gain more insight into the effect of supervisors’ abusive supervision on creativity.

## Data Availability Statement

The raw data supporting the conclusions of this article will be made available by the authors, without undue reservation.

## Ethics Statement

Ethical review and approval was not required for the study on human participants in accordance with the local legislation and institutional requirements. The patients/participants provided their written informed consent to participate in this study.

## Author Contributions

CS provided the idea, designed this study, and wrote the manuscript. JY and SH contributed to the research design, data collection and analysis, and manuscript revision. All authors contributed to the article and approved the submitted version.

## Conflict of Interest

The authors declare that the research was conducted in the absence of any commercial or financial relationships that could be construed as a potential conflict of interest.

## References

[B1] Aiken L. S. West S. G. Reno R. R. (1991). *Multiple Regression: Testing and Interpreting Interactions.* Thousand Oaks, CA: Sage.

[B2] Aryee S. Chen Z. X. Sun L. Y. Debrah Y. A. (2007). Antecedents and outcomes of abusive supervision: test of a trickle-down model. *J. Appl. psychol.* 92 191–201. 10.1037/0021-9010.92.1.191 17227160

[B3] Baron R. M. Kenny D. A. (1986). The moderator–mediator variable distinction in social psychological research: Conceptual, strategic, and statistical considerations. *J. Pers. Soc. Psychol.* 51 1173–1182. 10.1037/0022-3514.51.6.1173 3806354

[B4] Binnewies C. Ohly S. Niessen C. (2008). Age and creativity at work: the interplay between job resources, age andidea creativity. *J. Manag. Psychol.* 23 438–457. 10.1108/02683940810869042

[B5] Cerne M. Jaklic M. Skerlavaj M. (2013). Authentic leadership, creativity, and innovation: a multilevel perspective. *Leadership* 9 63–85. 10.1177/1742715012455130

[B6] Derue D. S. Ashford S. J. (2010). Who will lead and who will follow? A social process of leadership identity construction in organizations. *Acad. Manag. Rev.* 35 627–647. 10.5465/amr.35.4.zok627

[B7] Dong Y. Bartol K. M. Zhang Z. X. Li C. (2017). Enhancing employee creativity via individual skill development and team knowledge sharing: Influences of dual-focused transformational leadership. *J. Organ. Behav.* 38 439–458. 10.1002/job.2134

[B8] Epitropaki O. Kark R. Mainemelis C. Lord R. G. (2017). Leadership and followership identity processes: a multilevel review. *Leadership Q.* 28 104–129. 10.1016/j.leaqua.2016.10.003

[B9] Farh C. I. Chen Z. (2014). Beyond the individual victim: Multilevel consequences of abusive supervision in teams. *J. Appl. Psychol.* 99 1074–1095. 10.1037/a0037636 25111251

[B10] Farmer S. M. Tierney P. (2017). *Considering Creative Self-Efficacy: Its Current State and Ideas for Future Inquiry: The Creative Self.* Amsterdam: Elsevier, 23–47.

[B11] Farmer S. M. Tierney P. Kung-Mcintyre K. (2003). Employee creativity in taiwan: an application of role identity theory. *Acad. Manag. J.* 46 618–630. 10.5465/30040653 30040653

[B12] Feng J. Zhang Y. Liu X. Zhang L. Han X. (2018). Just the right amount of ethics inspires creativity: a cross-level investigation of ethical leadership, intrinsic motivation, and employee creativity. *J. Bus. Ethics* 153 645–658. 10.1007/s10551-016-3297-1

[B13] Festinger L. (1954). A theory of social comparison processes. *Hum. Relat.* 7 117–140. 10.1177/001872675400700202

[B14] Gerber J. Wheeler L. Suls J. (2018). A social comparison theory meta-analysis 60+ years on. *Psychol. Bull.* 144 177–197. 10.1037/bul0000127 29144145

[B15] Gong Y. Huang J. C. Farh J. L. (2009). Employee learning orientation, transformational leadership, and employee creativity: the mediating role of employee creative self-efficacy. *Acad. Manag. J.* 52 765–778. 10.5465/amj.2009.43670890

[B16] Gu Q. Tang T. L.-P. Jiang W. (2015). Does moral leadership enhance employee creativity? Employee identification with leader and leader–member exchange (LMX) in the Chinese context. *J. Bus. Ethics* 126 513–529. 10.1007/s10551-013-1967-9

[B17] Han G. H. Harms P. Bai Y. (2017). Nightmare bosses: The impact of abusive supervision on employees’ sleep, emotions, and creativity. *J. Bus. Ethics* 145 21–31. 10.1007/s10551-015-2859-y

[B18] Harris T. B. Li N. Boswell W. R. Zhang X. A. Xie Z. (2013). Getting what’s new from newcomers: empowering leadership, creativity, and adjustment in the socialization context. *Pers. Psychol.* 67 567–604.

[B19] Hirst G. Dick R. V. Knippenberg D. V. (2009). A social identity perspective on leadership and employee creativity. *J. Organ. Behav.* 30 963–982. 10.1002/job.600

[B20] Hu J. Liden R. C. (2013). Relative leader–member exchange within team contexts: How and when social comparison impacts individual effectiveness. *Pers. Psychol.* 66 127–172. 10.1111/peps.12008

[B21] James L. R. (1982). Aggregation bias in estimates of perceptual agreement. *J. Appl. Psychol.* 67:219 10.1037/0021-9010.67.2.219

[B22] Jiang W. Gu Q. Tang T. L.-P. (2017). Do victims of supervisor bullying suffer from poor creativity? Social cognitive and social comparison perspectives. *J. Bus. Ethics* 157 1–20.

[B23] Kaplan A. Garner J. K. (2017). A complex dynamic systems perspective on identity and its development: the dynamic systems model of role identity. *Dev. Psychol.* 53 2036–2051. 10.1037/dev0000339 29094968

[B24] Lee S. Yun S. Srivastava A. (2013). Evidence for a curvilinear relationship between abusive supervision and creativity in South Korea. *Leader. Q.* 24 724–731. 10.1016/j.leaqua.2013.07.002

[B25] Liao Z. Yam K. C. Johnson R. E. Liu W. Song Z. (2018). Cleansing my abuse: a reparative response model of perpetrating abusive supervisor behavior. *J. Appl. Psychol.* 103 1039–1056. 10.1037/apl0000319 29722999

[B26] Lin W. Ma J. Zhang Q. Li J. C. Jiang F. (2018). How is benevolent leadership linked to employee creativity? The mediating role of leader–member exchange and the moderating role of power distance orientation. *J. Bus. Ethics* 152 1099–1115. 10.1007/s10551-016-3314-4

[B27] Liu D. Gong Y. Zhou J. Huang J.-C. (2017). Human resource systems, employee creativity, and firm innovation: the moderating role of firm ownership. *Acad. Manag. J.* 60 1164–1188. 10.5465/amj.2015.0230

[B28] Liu D. Liao H. Loi R. (2012). The dark side of leadership: a three-level investigation of the cascading effect of abusive supervision on employee creativity. *Acad. Manag. J.* 55 1187–1212. 10.5465/amj.2010.0400

[B29] Liu W. Zhang P. Liao J. Hao P. Mao J. (2016). Abusive supervision and employee creativity: the mediating role of psychological safety and organizational identitification. *Manag. Dec.* 54 130–147. 10.1108/md-09-2013-0443

[B30] Luo Z. Wang Y. Marnburg E. Øgaard T. (2016). How is leadership related to employee self-concept? *Int. J. Hospital. Manag.* 52 24–32. 10.1016/j.ijhm.2015.09.003

[B31] Mackey J. D. Frieder R. E. Brees J. R. Martinko M. J. (2017). Abusive supervision: a meta-analysis and empirical review. *J. Manag.* 43 1940–1965. 10.1177/0149206315573997

[B32] Muller D. Judd C. M. Yzerbyt V. Y. (2005). When moderation is mediated and mediation is moderated. *J. Pers. Soc. Psychol.* 89 852. 10.1037/0022-3514.89.6.852 16393020

[B33] Murphy S. E. Ensher E. A. (2008). A qualitative analysis of charismatic leadership in creative teams: the case of television directors. *Leader. Q.* 19 335–352. 10.1016/j.leaqua.2008.03.006

[B34] Ng T. W. Feldman D. C. (2013). A meta-analysis of the relationships of age and tenure with innovation-related behaviour. *J. Occupat. Organ. Psychol.* 86 585–616.

[B35] Oyserman D. (2007). Social identity and self-regulation. *Soc. Psychol.* 2 432–453.

[B36] Peng A. C. Schaubroeck J. M. Li Y. (2014). Social exchange implications of own and coworkers’ experiences of supervisory abuse. *Acad. Manag. J.* 57 1385–1405. 10.5465/amj.2012.0080

[B37] Preacher K. J. Rucker D. D. Hayes A. F. (2007). Addressing moderated mediation hypotheses: theory, methods, and prescriptions. *Multiv. Behav. Res.* 42 185–227. 10.1080/00273170701341316 26821081

[B38] Priesemuth M. Schminke M. Ambrose M. L. Folger R. (2014). Abusive supervision climate: a multiple-mediation model of its impact on group outcomes. *Acad. Manag. J.* 57 1513–1534. 10.5465/amj.2011.0237

[B39] Qu R. Janssen O. Shi K. (2015). Transformational leadership and follower creativity: the mediating role of follower relational identification and the moderating role of leader creativity expectations. *Leader. Q.* 26 286–299. 10.1016/j.leaqua.2014.12.004

[B40] Stobbeleir K. E. M. D. Ashford S. J. Buyens D. (2011). Self-Regulation of creativity at work: the role of feedback-seeking behavior in creative performance. *Acad. Manag. J.* 54 811–831. 10.5465/amj.2011.64870144

[B41] Stoltzfus G. Nibbelink B. L. Vredenburg D. Hyrum E. (2011). Gender, gender role, and creativity. *Soc. Behav. Pers.* 39 425–432.

[B42] Strickhouser J. E. Zell E. (2015). Self-evaluative effects of dimensional and social comparison. *J. Exp. Soc. Psychol.* 59 60–66. 10.1016/j.jesp.2015.03.001

[B43] Tepper B. J. (2000). Consequences of abusive supervision. *Acad. Manag. J.* 43 178–190. 10.2307/1556375

[B44] Tepper B. J. Simon L. Park H. M. (2017). Abusive supervision. *Ann. Rev. Organ. Psychol. Organ. Behav.* 4 123–152.

[B45] Tierney P. Farmer S. M. (2011). Creative self-efficacy development and creative performance over time. *J. Appl. Psychol.* 96 277–293. 10.1037/a0020952 20954756

[B46] Tse H. H. Lam C. K. Lawrence S. A. Huang X. (2013). When my supervisor dislikes you more than me: the effect of dissimilarity in leader–member exchange on coworkers’ interpersonal emotion and perceived help. *J. Appl. Psychol.* 98:974. 10.1037/a0033862 23895039

[B47] Wang A. C. Cheng B. S. (2010). When does benevolent leadership lead to creativity? The moderating role of creative role identity and job autonomy. *J. Organ. Behav.* 31 106–121. 10.1002/job.634

[B48] Wang C. J. Tsai H. T. Tsai M. T. (2014). Linking transformational leadership and employee creativity in the hospitality industry: the influences of creative role identity, creative self-efficacy, and job complexity. *Tourism Manag.* 40 79–89. 10.1016/j.tourman.2013.05.008

[B49] Zhang H. Kwan H. K. Zhang X. Wu L.-Z. (2014). High core self-evaluators maintain creativity: a motivational model of abusive supervision. *J. Manag.* 40 1151–1174. 10.1177/0149206312460681

[B50] Zhang S. Ke X. Frank Wang X. H. Liu J. (2018). Empowering leadership and employee creativity: a dual-mechanism perspective. *J. Occup. Organ. Psychol.* 91 896–917. 10.1111/joop.12219

[B51] Zhang X. Bartol K. M. (2010). Linking empowering leadership and employee creativity: The influence of psychological empowerment, intrinsic motivation, and creative process engagement. *Acad. Manag. J.* 53 107–128. 10.5465/amj.2010.48037118

[B52] Zhou J. Hoever I. J. (2014). Research on workplace creativity: a review and redirection. *Ann. Rev. Organ. Psychol. Organ. Behav.* 1 333–359. 10.1146/annurev-orgpsych-031413-091226

